# Landslide Displacement Prediction Based on Time Series Analysis and Double-BiLSTM Model

**DOI:** 10.3390/ijerph19042077

**Published:** 2022-02-12

**Authors:** Zian Lin, Xiyan Sun, Yuanfa Ji

**Affiliations:** 1 School of Computer Science and Information Security, Guilin University of Electronic Technology, Guilin 541004, China; 20031102010@mails.guet.edu.cn; 2 Guangxi Key Laboratory of Precision Navigation Technology and Application, Guilin University of Electronic Technology, Guilin 541004, China; jiyuanfa@163.com; 3 Information and Communication School, Guilin University of Electronic Technology, Guilin 541004, China; 4 GUET-Nanning E-Tech Research Institute Co., Ltd., Nanning 530031, China

**Keywords:** landslide displacement prediction, bidirectional long short term memory, time series analysis, maximum information coefficient

## Abstract

In recent years, machine learning models facilitated notable performance improvement in landslide displacement prediction. However, most existing prediction models which ignore landslide data at each time can provide a different value and meaning. To analyze and predict landslide displacement better, we propose a dynamic landslide displacement prediction model based on time series analysis and a double-bidirectional long short term memory (Double-BiLSTM) model. First, the cumulative landslide displacement is decomposed into trend and periodic displacement components according to time series analysis via the exponentially weighted moving average (EWMA) method. We consider that trend displacement is mainly influenced by landslide factors, and we apply a BiLSTM model to predict landslide trend displacement. This paper analyzes the internal relationship between rainfall, reservoir level and landslide periodic displacement. We adopt the maximum information coefficient (MIC) method to calculate the correlation between influencing factors and periodic displacement. We employ the BiLSTM model for periodic displacement prediction. Finally, the model is validated against data pertaining to the Baishuihe landslide in the Three Gorges, China. The experimental results and evaluation indicators demonstrate that this method achieves a better prediction performance than the classical prediction methods, and landslide displacement can be effectively predicted.

## 1. Introduction

Landslides are common geological disasters worldwide. Landslides not only damage the natural environment, cause soil erosion on slopes and alter landforms but also destroy buildings and infrastructure in villages and towns and result in a large number of casualties in serious cases. China is one of the countries most seriously affected by landslide disasters due to its vast territory, complex terrain and changeable climate. In China, many people live and work on and near the slopes. Although a landslide occurs suddenly, the process of a slope becoming a landslide can be monitored and predicted to a certain extent. An accurate landslide displacement prediction model combined with a landslide warning model can effectively improve people’s judgment of landslides in daily life and help decision makers make more accurate decisions and protect people’s life and health, so as to achieve the purpose of improving population wellbeing. According to China’s national geological disaster report for 2020, 4810 landslides occurred in China in 2020, accounting for 61.3% of all geological disasters in China, resulting in a large number of property losses and casualties [1]. Local landslide events were initially recorded when China built the world’s largest hydropower station, the Three Gorges Dam, in 2003 [2,3]. The environment of the Three Gorges Reservoir is highly conducive to the formation of landslides in terms of the topography, geology and climate [4]. The Three Gorges Project has faced intense pressure in terms of geological disaster prevention [2]. This paper aims to help the Three Gorges Project and similar engineering projects with regard to geological disaster prevention. Therefore, it is particularly important and necessary to predict landslide displacement in this area.

Landslide displacement prediction models generally include physical and data-based models [3,5,6]. Because of the complexity of landslides, the acquisition of a physical model of landslide displacement before landslide occurrence is very difficult. [7]. There are many kinds of physics-based models. Jiang et al. [8] simulated landslide displacement based on fluid-solid coupling theory. Herrera et al. [9] adopted a physics-based one-dimensional infinite viscoplastic model to predict Portalet landslide motion. Mufundirwa et al. [10] considered physical characteristics to evaluate the validity of the inverse velocity (INV) and evaluated this parameter in the laboratory to predict rock mass destruction attributed to landslides. Due to the complexity of geological conditions, physics-based models cannot simulate the actual structure of the geographical environment well, and the prediction performance can therefore be affected by both known and unknown factors, leading to inevitable errors in landslide displacement prediction. Moreover, landslides are difficult to predict in a timely and accurate manner. Even if the same physics-based landslide displacement prediction model is applied to different landslides, the physical characteristics are inconsistent due to the varying landslide conditions, which can lead to different prediction accuracies.

In recent years, data-based models have become more popular than physics-based models. Data-based models usually treat landslides as nonlinear systems [6,11]. Landslide occurrence is caused by many reasons. There are many internal reasons determining landslide displacement, such as the topography, landslide structure, rock and soil properties and other internal geological factors. External causes usually include the joint action of reservoir water level change, rainfall, snow melt and other factors. Many data-based models divide landslide displacement into trend and period displacement terms by analyzing the composition of landslide displacement, thereby predicting the trend and period displacement term, respectively. Finally, the predicted trend and period terms are added to obtain the final predicted landslide displacement [12,13]. Huang et al. [3] and Huang et al. [6] decomposed landslide displacement according to chaos theory and then combined this approach with an extreme learning machine (ELM) model to predict landslide displacement. Due to the complexity of landslides and model limitations, it is difficult to describe the deformation and evolution of landslides accurately with a single model [14]. Therefore, based on the principle of the optimal weight, Li et al. [14] combined the GM(1,1) and Verhulst models, retained the advantages of these two models and obtained a better combined model to predict landslide displacement. A hybrid model comprising support vector regression (SVR) and a long short term memory (LSTM) network was employed; the cost function and penalty mechanism were proposed, and improved gray wolf optimization was implemented to determine the connection parameters of the hybrid model [15].

Because landslide displacement entails a kind of typical time series data, time series analysis methods are often applied to analyze landslide displacement and construct landslide prediction models [12,16,17]. Landslide prediction models generally do not predict a single value, but these models predict a landslide displacement range, which determines the prediction accuracy [18,19]. Xie et al. [20] considered conditions such as landslide profiles, rock properties of landslides, slopes and land use properties and applied the LSTM model to predict landslide displacement directly. Zhang et al. [21] adopted the gated recurrent unit (GRU) model to predict landslide displacement. There are also many studies that decompose landslide displacement according to different frequencies. Xing et al. [22] applied variational mode decomposition (VMD) to decompose landslide displacement into multidimensional signals, while Lian et al. [23] and Lian et al. [24] conducted ensemble empirical mode decomposition (EEMD) to decompose original landslide sequence data into several subsequences with distinct frequencies. Xu et al. [25] implemented empirical mode decomposition (EMD) to decompose landslide data. The original data were interpolated to increase the scale of the model training set to improve the prediction accuracy. Because there are many factors influencing landslides, many studies adopt the correlation method to calculate these factors, while factors with a high correlation are selected, and factors with a low correlation are eliminated to improve the model prediction performance. Pearson’s cross-correlation coefficients (PCCs) and mutual information (MI) correlation coefficients are considered to calculate the correlation between landslide variables and landslides [26]. The maximum information coefficient (MIC) method was adopted to select model input variables [27]. The gray relational degree (GRD) method was applied to explain which data could be employed as input variables [28]. The Gini coefficient was determined to quantify the importance of influencing factors [29]. Yang et al. [16] and Zhou et al. [30] not only applied the monthly precipitation and reservoir water level as input variables in periodic displacement prediction but also applied the trend displacement component and previous precipitation and reservoir water levels to improve the prediction accuracy of landslide periodic displacement effectively.

Time series data are data collected at different times; they can describe phenomenon changes over time. This kind of data can reflect the state or extent to which something has changed over time [31]. Landslide displacement data are such a kind of data which can reflect the stability and changing state of the landslide itself with time change. However, previous research results did not comprehensively treat landslide displacement data as time series data but as ordinary data, or only conducted time series analysis of the period term in displacement data. Previous studies further ignored that landslide displacement data, as a data series that changes over time, provide varying meanings at different time points. If the time series data are treated as ordinary numbers, the time relationship between the data will be ignored [31].

This paper considers that landslide displacement data are typical time series data, and a hybrid dynamic landslide displacement prediction model based on data is constructed by combining a time series analysis method and deep learning model. Then, we use the established hybrid dynamic model to simulate the Baishuihe landslide in the Three Gorges and successfully predict the feasibility and effectiveness of the model.

The main contributions of this paper are as follows:

1. Attaching importance to landslide data is also the time series data. The exponentially weighted moving average (EWMA) method is applied to decompose actual landslide displacement data, which endows recent data with a higher significance and increases the weight in the data decomposition process.
2. Considering that the trend displacement component of landslide displacement also comprises typical time series data, the BiLSTM model is adopted to predict landslide trend displacement.
3. This paper analyzes the internal relationship between rainfall, reservoir water level and landslide periodic displacement. The MIC method is adopted to calculate the correlation between each influencing factor and periodic displacement, and 11 influencing factors are obtained that are highly correlated with periodic displacement. Due to the periodicity and repeatability of rainfall and reservoir water level changes in the Baishuihe landslide area, the bidirectional long short term memory (BiLSTM) model is trained with the identified highly correlated factors, and the periodic term of landslide displacement is predicted. The final predicted landslide displacement constitutes the sum of the trend term predicted with the BiLSTM model and the period term predicted with the BiLSTM model.

The remainder of this paper is organized as follows: the second part presents the basic principle of the prediction model based on time series analysis and the dynamic hybrid Double-BiLSTM model. In Section 3, we introduce a practical application case to verify the proposed hybrid dynamic prediction model. The validity and accuracy of the prediction model are verified via experiments. In Section 4, we evaluate the model based on specific evaluation metrics and discuss the model limitations and future improvement plans. The last section provides the conclusion of this paper.

## 2. Materials and Methods

### 2.1. Time Series Analysis of Landslide Displacement

Landslide displacement refers to the distance that the soil or rock mass on the slope slides down the slope as a whole or separately under the action of gravity under the influence of river erosion, groundwater activity, rainwater immersion, earthquake and artificial slope cutting. The generation of landslide displacement is influenced by both the internal geological conditions (geological structure, landform, lithology, etc.) and external influencing factors (rainfall, reservoir water level, etc.) of a given landslide location [2,6,32]. Under the influence of internal geological conditions, landslide displacement exhibits an approximate increasing function over time. External factors influence landslide displacement via seasonal changes and generate periodic changes. Based on the above information, this paper considers time series analysis theory to decompose the actual landslide displacement into three parts: linear trend displacement, nonlinear periodic displacement and random displacement [12,33]. Trend displacement is influenced by internal factors such as topography and soil properties. Periodic displacement is affected by rainfall, groundwater and reservoir level changes. Random displacement is determined by random loads such as wind load or random rainfall processes such as nonseasonal rainfall and temporary changes in reservoir water level. Therefore, the landslide cumulative displacement can be expressed as Equation (1).

(1) D=T+P+N

where D is the landslide cumulative displacement; T is the landslide trend displacement; P is the landslide periodic displacement; and N is the landslide random displacement. However, because random displacement is not regularly generated, it cannot be reasonably and accurately predicted. To facilitate analysis, random displacement is usually ignored, and the landslide cumulative displacement is considered to be produced and developed under the joint action of geotechnical conditions and external influencing factors [12,16,33]. Therefore, the cumulative displacement can be decomposed into deterministic trend displacement reflecting the slope rock and soil conditions and nonlinear periodic displacement under the action of external factors. This paper is therefore based on the above theory. Then, the cumulative displacement can be defined as Equation (2).

(2) D=T+P

### 2.2. Exponentially Weighted Moving Average Method

Landslide trend displacement can reflect the long-term development trend of the rock and soil conditions of landslides. In most previous studies, the ordinary moving average (MA) method was employed to decompose historical landslide displacement into trend and periodic displacement components [18,25]. However, the MA method does not consider the importance of the data pertaining to each period and considers that the data at each time point are equally important. This paper proposes that the time series data pertaining to different periods provide varying time values and employs the EWMA method to compensate for the ordinary MA method due to the lack of observation data. Moreover, according to the characteristics whereby recent data exert a greater impact on prediction values, a higher weight is assigned to recent data, and a lower weight is assigned to remote data. The EWMA method can be expressed as Equation (3).

(3) $$T_{t}=\alpha D_{t-1}+\left(1-\alpha \right)T_{t-1}$$

where T denotes trend displacement; D denotes the landslide displacement data; and α is a constant term between 0 and 1, reflecting the rate of weighted decline. Xiu et al. [34] reported that when α varies between 0.05 and 0.3, slight changes can be detected more effectively. After many experiments, this article chooses α = 0.25.

### 2.3. Maximum Information Coefficient Method

Reshef et al. [35] proposed a new metric based on information theory, namely, the MIC, in 2011. The MIC can measure the dependence between different variables and effectively considers nonfunctional dependence, which cannot be represented by a single function. The MIC mainly relies on mutual information and grid partitioning methods for calculation. MI is an index to measure the level of correlation between variables. It is assumed that variable $A=\left\{a_{i},\;i=1,\;2,\ldots ,n\right\}$ and variable $B=\left\{b_{i},\;i=1,\;2,\ldots ,n\right\}$, where n is the number of samples, and MI is defined as Equation (4).

(4) $$MI\left(A,B\right)=\underset{a\in A\;}{\sum }\underset{b\in B}{\sum }p\left(a,b\right)\log\frac{p\left(a,b\right)}{p\left(a\right)p\left(b\right)}$$

where p(a) is the marginal probability density of A; p(b) is the marginal probability density of B; and p(a,b) is the joint probability density of A and B. Assuming that $D=\left\{\left(a_{i},b_{i}\right),\;i=1,\;2,\ldots ,n\right\}$ comprises a finite set of ordered pairs, G is defined to divide the range of A into x segments and the range of B into y segments, and G denotes the x×y grid. MI(A,B) is calculated in each grid partition. There are multiple grids of the same  x×y dimensions, and the MI value of G is obtained based on the maximum MI(A,B) value among the different grids. The maximum MI equation of D under partition G is defined as Equation (5).

(5) $$MI^{*}\left(D,x,y\right)=\max\;MI(D|G)$$

where D|G denotes that data D are partitioned using G. Although the MIC method relies on MI to represent the grid quality, it does not simply estimate MI. The maximum normalized MI values obtained under the different partitions comprise an eigenmatrix, which is defined as $MI{\left(D\right)}_{x,y}$, and this eigenmatrix can be defined as Equation (6).

(6) $$MI{\left(D\right)}_{x,y}=\frac{MI^{*}\left(D,x,y\right)}{\log\left(\min\left\{x,y\right\}\right)}$$

The MIC equation is expressed as Equation (7).

(7) $$MIC\left(D\right)=\underset{xy<B\left(n\right)}{\max}\left\{M{\left(D\right)}_{x,y}\right\}$$

where B(n) denotes the upper bounds of grid partition x×y. Xiu et al. (2020) determined that the effect is optimal for B(n) = n0.6. Therefore, this value is also applied in the experimental simulations.

### 2.4. Bidirectional Long Short Term Memory Neural Network Model

The LSTM model is a unique recurrent neural network (RNN) employed to solve the problems of gradient disappearance and gradient explosion of original RNNs and was proposed by Hochreiter and Schmidhuber [36] in 1997. Because of its special memory and gate structures, this model can better learn the correlation features contained in time series data, so the LSTM model is widely adopted in time series prediction models. Yang et al. [16] and Xie et al. [20] employed the LSTM model to predict landslide periodic displacement directly in view of the characteristics whereby the LSTM model suitably processes time series data. Xing et al. [15] combined the LSTM and SVR models, retained the advantages of these two models, improved the model generalization ability and predicted landslide periodic displacement. However, there is no research on landslide trend displacement prediction with LSTM models. The basic unit of the LSTM model includes the forget, input and output gates.

**Figure 1:**
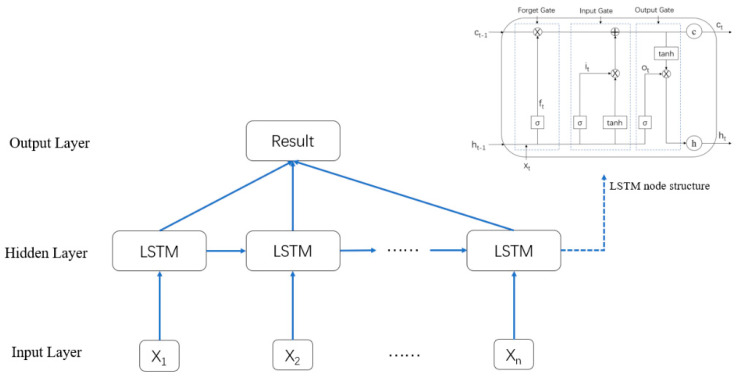
Long short term memory model architecture.

Each gate exhibits a unique function. In the forget gate, the input $\;x_{t}$, state memory unit $C_{t-1}$ and intermediate output $h_{t-1}$ jointly determine the forgetting part of the state memory unit. In the input gate, the result of $x_{t}$, which is calculated with the sigmoid and tanh functions, determines the reserved information in the state memory unit. In the output gate, the intermediate output $h_{t}$ is determined jointly by the updated $C_{t}$ and output $o_{t}$. The equations for the unit are expressed as Equations (8)–(13).

(8) $$f_{t}=\sigma \left(w_{f}\cdot \left[h_{t-1},x_{t}\right]+b_{f}\right)$$

(9) $$i_{t}=\sigma \left(w_{i}\cdot \left[h_{t-1},x_{t}\right]+b_{i}\right)$$

(10) $$o_{t}=\sigma \left(w_{o}\cdot \left[h_{t-1},x_{t}\right]+b_{o}\right)$$

(11) $$C_{t}=f_{t}\cdot C_{t-1}+i_{t}\cdot {\tilde{C}}_{t}$$

(12) $$h_{t}=o_{t}\cdot \tan h\left(C_{t}\right)$$

(13) $${\tilde{C}}_{t}=\tan h\left(W_{c}\cdot \left[h_{t-1},x_{t}\right]+b_{c}\right)$$

where $x_{t}$ denotes the input data; $f_{t}$ denotes the forget gate; $i_{t}$ denotes the input gate; $o_{t}$ denotes the output gate; $h_{t}$ denotes the output data; $C_{t}$ denotes the state of the cell; ${\tilde{C}}_{t}$ denotes the temporary state of the cell; $w_{f}$ denotes the weight of the forget gate; $w_{o}$ denotes the weight of the output gate; $w_{i}$ denotes the weight of the input gate; $w_{c}$ denotes the weight of the temporary state; $b_{f}$ denotes the bias of the forget gate; $b_{i}$ denotes the bias of the input gate; $b_{o}$ denotes the bias of the output gate; $b_{c}$ denotes the bias of the temporary state; σ denotes the sigmoid function; tanh denotes the tanh function; [] denotes the connection between two vectors; and · denotes the matrix product. Figure 1 shows the LSTM architecture.

**Figure 2:**
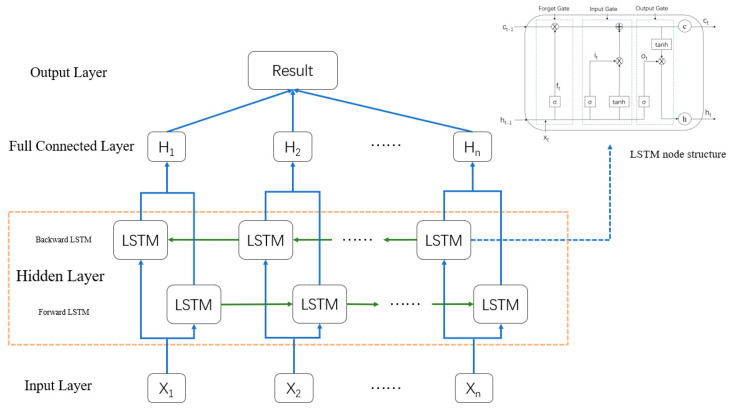
Bidirectional long short term memory model architecture.

Although the LSTM model solves the gradient disappearance or gradient explosion problem of RNNs, the LSTM model can only learn previous information and cannot consider future information. Because the factors influencing landslide periodic displacement are repeatable and periodic, with regard to the training model, landslide periodic displacement is related not only to historical periodic displacement information but also to future information, which can play an auxiliary role in model training. Therefore, the BiLSTM model was applied in this paper instead of the LSTM model to predict landslide trend displacement and periodic displacement, which not only solved the gradient disappearance or gradient explosion problem encountered in the model but also made it easier to learn the dependence between the historical and future displacement data at the different time points. In addition, the displacement characteristics obtained were global. The BiLSTM model fully considers the data information before and after the current displacement data during model training. At present, the BiLSTM model has been successfully applied in many prediction fields, including solar radiation hourly prediction [37], well log prediction [38] and tourism demand prediction [39]. Figure 2 shows the BiLSTM model structure adopted in this paper.

The BiLSTM model is a combination of forward and reverse LSTM models. The forward LSTM model obtains historical information from the current time point displacement data from left to right (forward), while the reverse LSTM model extracts future information from the current time point displacement data along the opposite direction. Hence, this can be expressed as Equation (14):

(14) $$H_{t}=\left[\vec{h}{\;}_{t},\overset{\leftarrow }{h}{\;}_{t}\right]$$

where ${\overset{\leftarrow }{h}}_{t}$ is the state of the reverse LSTM output; ${\vec{h}}_{t}$ is the state of the forward LSTM output; $H_{t}$ is the state of the BiLSTM output; and [] denotes the connection between two vectors. Splicing the hidden layer vectors obtained along the forward and reverse directions and mapping through the fully connected layer, a one-dimensional vector is obtained as the output of the BiLSTM layer, namely, the landslide trend displacement and periodic displacement to be predicted.

### 2.5. Double-BiLSTM Model

Because the trend displacement and periodic displacement of the landslide are typical time series data, the trend displacement reflects the process of the slope’s own properties changing with time, and the periodic displacement reflects the process of the slope’s external influence factors changing with time. Therefore, the BiLSTM model is used in this paper to predict trend displacement and periodic displacement, respectively. According to Formula 2, the final predicted displacement of the landslide is the synthesis of landslide trend displacement and periodic displacement; it is shown as

(15) $$D=T_{BiLSTM}+P_{BiLSTM}$$

where *D* is the final predicted displacement of the landslide; $T_{BiLSTM}$ is the predicted trend displacement of the landslide; and $P_{BiLSTM}$ is the predicted cycle displacement of the landslide.

### 2.6. Performance Indices

To evaluate the prediction performance of the proposed landslide displacement prediction model reasonably, specific performance indices were considered to evaluate the model prediction performance [32,40,41,42,43]. This paper introduces four evaluation indices, namely, the mean absolute error (MAE), root mean square error (RMSE), mean absolute percentage error (MAPE) and coefficient of determination R2 (R-square). In this paper, MAE is the average value of the absolute error between the predicted and real values, which describes the overall prediction performance. RMSE can measure the situation of mutation points in the predicted values. The effect of mutation points on RMSE is notable. MAPE considers not only the relationship between the predicted and actual values but also the error between the predicted and actual values. R2 can measure the capacity of the regression model and evaluate its fitting degree. The four evaluation indices are expressed as Equations (15)–(18).

(16) $$\mathrm{MAE}=\frac{1}{n}\underset{i=1}{\overset{n}{\sum }}\left|{\hat{y}}_{i}-y_{i}\right|$$

(17) $$\mathrm{RMSE}=\sqrt{\frac{1}{n}\underset{i=1}{\overset{n}{\sum }}{\left({\hat{y}}_{i}-y_{i}\right)}^{2}}$$

(18) $$\mathrm{MAPE}=\frac{100\%}{n}\underset{i=1}{\overset{n}{\sum }}\left|\frac{{\hat{y}}_{i}-y_{i}}{y_{i}}\right|$$

(19) $$R2=1-\frac{\underset{i=1}{\overset{n}{\sum }}{\left({\hat{y}}_{i}-y_{i}\right)}^{2}}{\underset{i=1}{\overset{n}{\sum }}{\left({\bar{y}}_{i}-y_{i}\right)}^{2}}$$

where *n* is the number of landslide data; $\hat{y}=\left\{{\hat{y}}_{1},\;{\hat{y}}_{2},\ldots ,{\hat{y}}_{n}\right\}$ denotes the predicted values of the model; $y=\left\{y_{1},y_{2},\ldots y_{n}\right\}$ denotes the measured values of the Baishuihe landslide; and $\bar{y}=\left\{{\bar{y}}_{1},\;{\bar{y}}_{2},\ldots ,{\bar{y}}_{n}\right\}$ denotes the average measured values of the Baishuihe landslide.

### 2.7. Landslide Displacement Prediction Process

**Figure 3:**
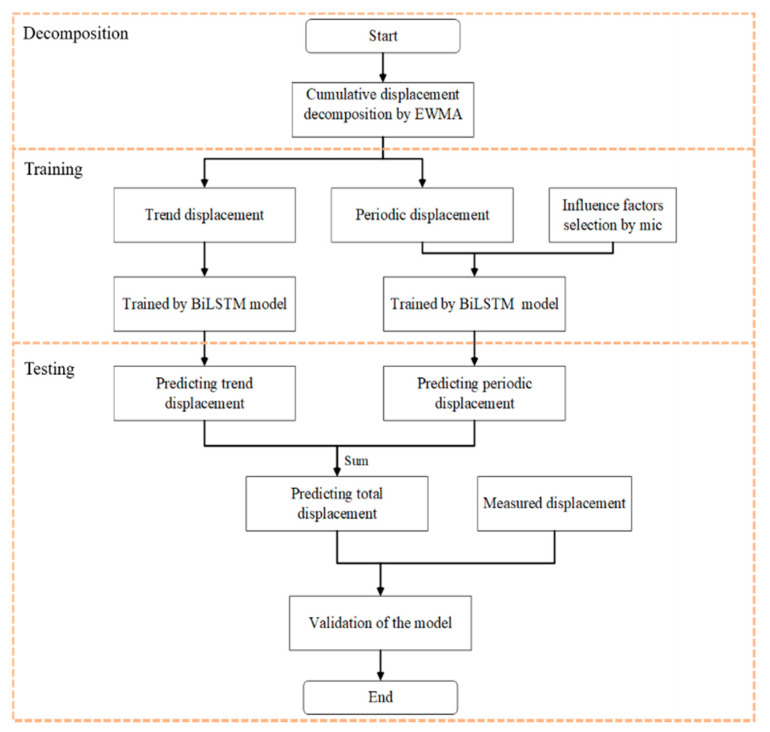
Flowchart of the landslide displacement prediction model.

Figure 3 shows the complete implementation process of the model proposed in this paper. Step 1: considering the characteristics of time series data, the EWMA method is applied to decompose the historical landslide displacement, and trend and periodic displacement components are obtained based on the historical displacement data. Step 2: the BiLSTM model is trained according to the historical trend displacement data, and the subsequent trend displacement is predicted. Step 3: the MIC is considered to calculate factors that may be intrinsically related to historical periodic displacement, and factors with a higher correlation are selected as input items of the BiLSTM model. Step 4: according to historical periodic displacement data and the selected influencing factors, the BiLSTM mixed prediction model is trained to predict subsequent periodic displacements. Step 5: the predicted cumulative displacement is obtained by adding the displacement results obtained in Steps 2 and 4 at the same time point. Step 6: the predicted cumulative displacement is compared to the actual cumulative displacement obtained via monitoring, and the predicted results are evaluated with evaluation indices.

## 3. Results

### 3.1. Baishuihe Landslide

**Figure 4:**
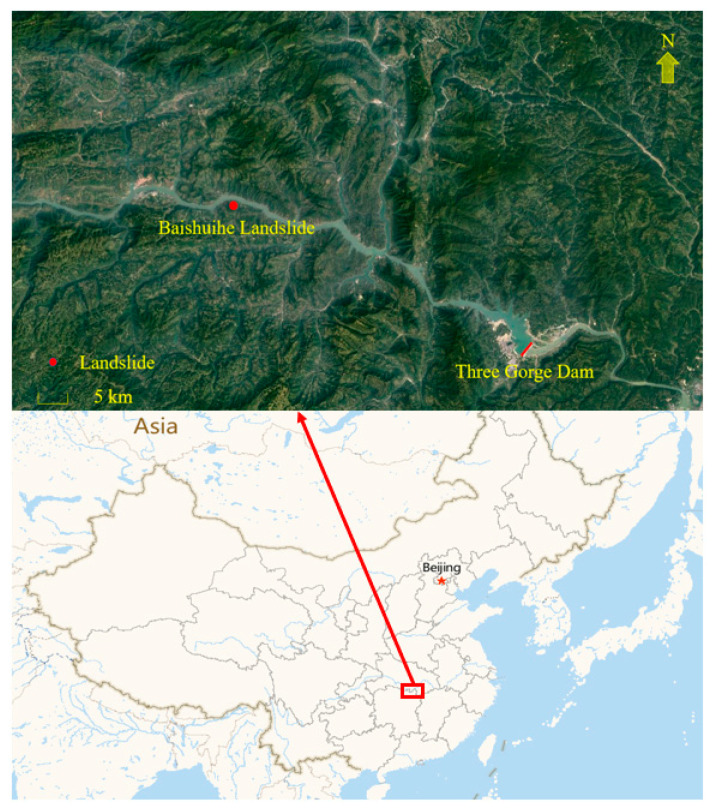
Geographical location of the Baishuihe landslide in the Three Gorges Reservoir area.

This paper analyzes the Baishuihe landslide, which is located near the Three Gorges Reservoir, to validate the time series analysis method and Double-BiLSTM hybrid dynamic landslide displacement prediction model. The specific geographical location of the Baishuihe landslide is shown in Figure 4.

The Baishuihe landslide is located on the south bank of the Yangtze River upstream of the Three Gorges Dam, with a longitude of 110°32’09” and latitude of 31°01’34”. The landslide is approximately 56 km from the Three Gorges Dam. The terrain exhibits a ladder-shaped topology, belonging to the Baishuihe village, Shazhenxi town, Zigui County. The top elevation of the slope ranges from 450 to 500 m. At an elevation from 180–500 m, the terrain slope ranges from 24 to 36°. From 130–180 m, the terrain is relatively flat with a slope ranging from 5 to 12°. At elevations from 80–130 m, the slope ranges from 27 to 31°. The overall leading and trailing edges of the terrain slope are relatively uneven and relatively gentle in the middle, resulting in a monocline slope.

There are obvious signs of displacement of the Baishuihe landslide. Since 2005, there occurred a partial tensile crack collapse at the back of the landslide. To date, the cracks on the east side and back edge of the landslide are basically transecting cracks; there are a large number of tensile cracks in the west, and local shallow failure is often produced.

**Figure 5:**
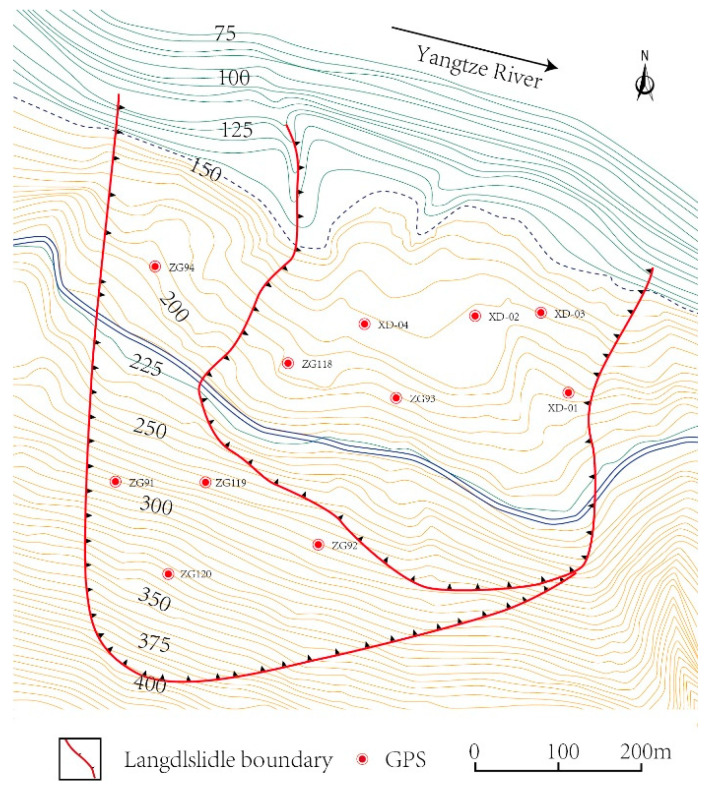
The installation position of GPS points.

The Baishuihe landslide contains a total of 11 global positioning system (GPS) points, among which ZG118 is located at the center of the landslide, which better reflects the process of landslide displacement change than do other GPS points. Xing et al. [15] and Miao et al. [32] also applied the monitoring data of ZG118 as experimental data in displacement prediction, so this paper also employs the data at the ZG118 monitoring point in experiments. The installation position of GPS points is shown in Figure 5.

**Figure 6:**
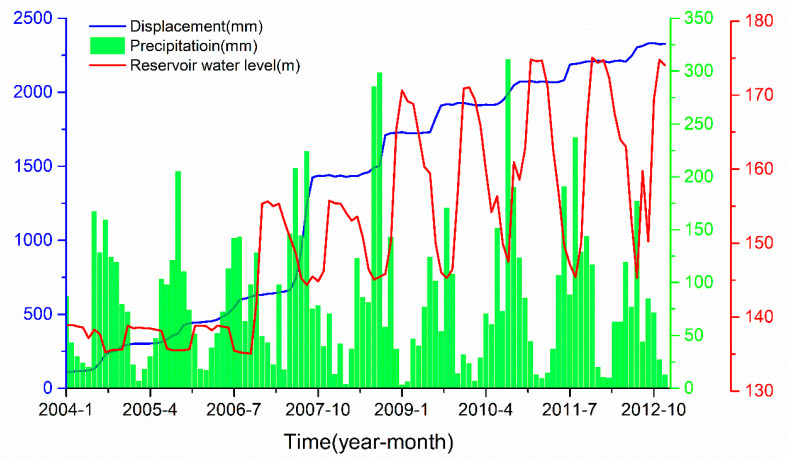
Displacement, rainfall and reservoir water level at location ZG118 in the Baishuihe landslide.

In this paper, 108 landslide displacement, rainfall and reservoir water level change data points pertaining to the Baishuihe landslide from January 2004 to December 2012 are considered. This dataset is provided by the National Cryosphere Desert Data Center/National Service Center for Speciality Environmental Crisis Observation. As shown in Figure 6, the data acquisition frequency is once a month. Through modeling and simulation of these data, the effectiveness of the EWMA algorithm proposed in this paper based on the principle of time series analysis and the feasibility and effectiveness of the Double-BiLSTM hybrid model are verified.

**Figure 7:**
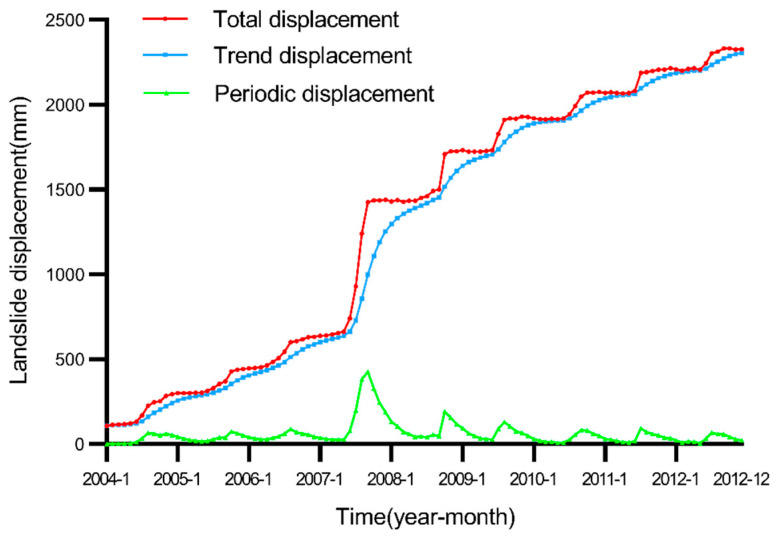
Displacement decomposition of the Baishuihe landslide.

According to time series analysis theory, the EWMA method is implemented to decompose the actual landslide displacement to obtain landslide trend and periodic displacement components. Xiu et al. [34] reported that when α changed between 0.05 and 0.3, small changes could be detected more effectively. Therefore, we conducted experiments with α values of 0.05, 0.1, 0.15, 0.2, 0.25 and 0.3, respectively. As the periodic displacement is mainly affected by external factors, the periodic displacement obtained by decomposition is correlated with rainfall and reservoir water level, and the results are shown in the following table:

As can be seen from the correlation results in Table 1, when α = 0.25, the periodic displacement is highly correlated with rainfall and reservoir water level, so we choose α = 0.25 to decompose landslide displacement in this paper. These three displacement components of the Baishuihe landslide are shown in Figure 7.

### 3.2. Trend Displacement Prediction

**Figure 8:**
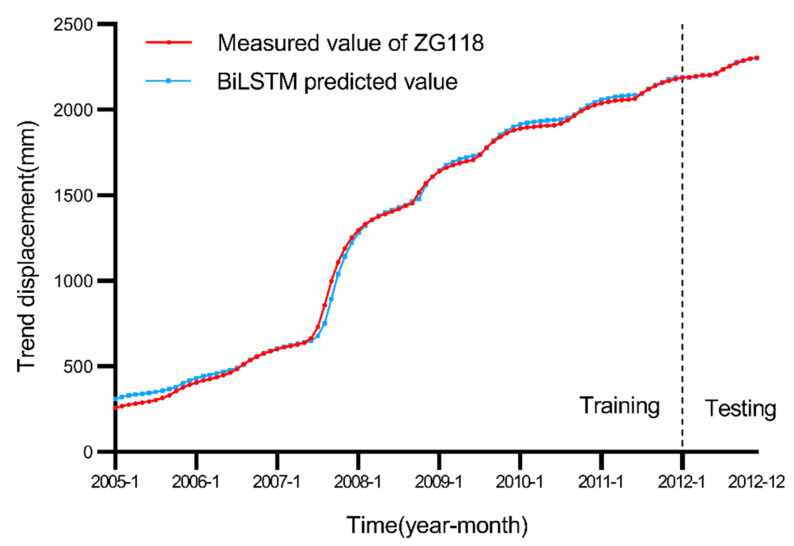
Comparison of the landslide trend displacement results.

Because landslide trend displacement is less affected by external factors and mainly influenced by internal factors, landslide displacement can feed back the current landslide state to a certain extent [16,30]. This paper intends to introduce trend displacement data one and two months before the landslide occurrence as the BiLSTM model input sequence into the training model to predict future trend displacements. A total of 108 months of Baishuihe landslide data was selected for the landslide trend displacement simulation experiment. The trend displacement data over the first 96 months were selected as the training set of the BiLSTM model, and the trend displacement data over the following 12 months were selected as the test set to verify the prediction performance of the BiLSTM model. Figure 8 shows that at the early stage of model training, the model fitting effect is not particularly good, and there occurs a certain deviation from the trend of the actual displacement, but with an increasing amount of data, the model fitting degree gradually increases. After 96 training data points, the prediction model trained on the test set clearly indicates that the BiLSTM model can effectively predict the trend displacement component of the Baishuihe landslide.

### 3.3. Periodic Displacement Prediction

Landslide periodic displacement differs from trend displacement, which is mainly influenced by external factors, while the rainfall and reservoir water levels in the Three Gorges area periodically fluctuate every year. Figure 6 shows that from April to August each year, when the rainfall sharply increases and the reservoir water level declines, the slope body becomes active, and the slope body increases the resultant landslide displacement. Conversely, landslides deform slowly at a constant rate. Therefore, it can be inferred that rainfall and the reservoir water level are closely related to landslide displacement [6,19]. During the rainy season, rainfall in the Baishuihe landslide area increases rapidly, and landslide displacement increases with a slight lag with increasing rainfall. In the rainy season, the landslide displacement range is 30–70 mm every month, and in the dry season, the range is 0–5 mm every month.

The influence of rainfall on landslides is multifaceted. On the one hand, the infiltration of rainfall into a given slope can lead to an increase in the weight of the slope, thus increasing the speed of the landslide. On the other hand, the impact of rainwater and water flow from high to low locations can affect the whole structure of the slope body, and the greater the impact of precipitation, the more likely a landslide occurs. In addition, rain infiltration can moisten soil, reduce friction between soil particles, and decrease the shear performance of sliding soil. Since the rainy season in the Baishuihe landslide area lasts for several months each year, and the dry season also lasts for several months, the impact of rainfall entails a persistent process acting on landslide displacement. Therefore, in addition to adopting the rainfall in the current month as an input variable to predict periodic displacement, this paper considers the rainfall in the previous month and rainfall in the previous two months as input variables to predict periodic displacement.

The slope body can occur in an extremely complicated state before landslide occurrence. When the slope occurs in a stable state, there are no obvious changes in the slope due to notable external influences, such as the rainy season with much precipitation and a long duration, an earthquake or a sharp decline in the reservoir water level. However, when the slope occurs in an unstable state, slight external factors can disrupt the primary balance, leading to landslide occurrence. Therefore, this paper intends to analyze the actual displacement one and two months before landslide occurrence and the displacement in the first two months to represent the state of the slope body indirectly, and these three variables are adopted as input variables for landslide periodic displacement.

With the arrival of the rainy season and the increase in precipitation, the dam reservoir can release water to ensure the safety of the dam due to the limited water capacity of the Three Gorges Dam, resulting in a sharp decline in the reservoir water level. When the reservoir water level decreases, the surface resistance of landslides is reduced, and the difficulty of landslides is reduced. The faster the reservoir water level decreases, the faster the increase in landslide displacement. Upon water discharge from the reservoir, water movement is accelerated, and the force generated also directly affects the stability of the slope. Figure 6 shows that the change in the reservoir water level exerts a certain lag effect on the Baishuihe landslide. Therefore, the reservoir water level one and two months before landslide occurrence and the change in reservoir water level in the first two months are considered in this paper as external factors influencing landslide displacement, and these factors are considered displacement input variables of the training cycle.

**Figure 9:**
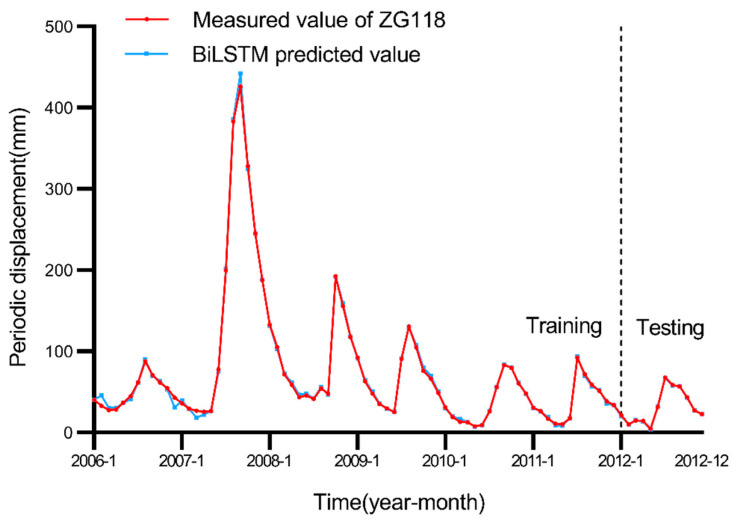
Comparison of the landslide periodic displacement results.

In this paper, the first 96 months of periodic displacement data of the Baishuihe landslide are selected as the training set of the model, and the subsequent 12 months of periodic displacement data are selected as the test set. In addition, the MIC method is applied to calculate the correlation between the influencing factors and periodic displacement of the Baishuihe landslide. The factors with a higher correlation are selected as the input sequence of the BiLSTM model, and the output sequence of the BiLSTM model comprises the predicted periodic displacement. The predicted results are shown in Figure 9, and it can be observed that the BiLSTM model can accurately predict the periodic displacement of the Baishuihe landslide.

### 3.4. Total Accumulated Displacement Prediction

**Figure 10:**
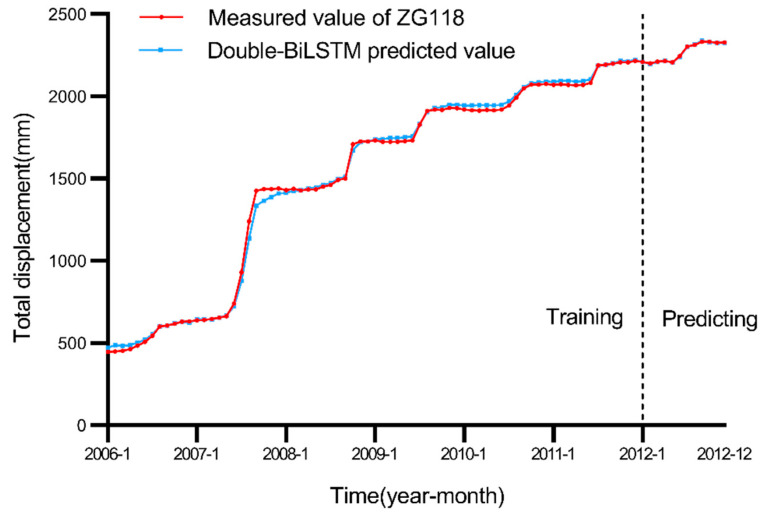
Comparison of the landslide total displacement results.

After predicting the trend and periodic displacement components of the Baishuihe landslide over the last 12 months, according to the EWMA-based landslide displacement decomposition principle (Equation (2)) proposed in this paper, the model prediction process is completed by adding the trend and periodic displacement values corresponding to these 12 months, and the predicted landslide displacement is thus obtained. As shown in Figure 10, the predicted results with the Double-BiLSTM model fluctuated with the actual data at the early training stage. After 96 months of data training, the predicted results obtained with the Double-BiLSTM model were remarkably close to the actual displacement values in the following 12 months.

**Figure 11:**
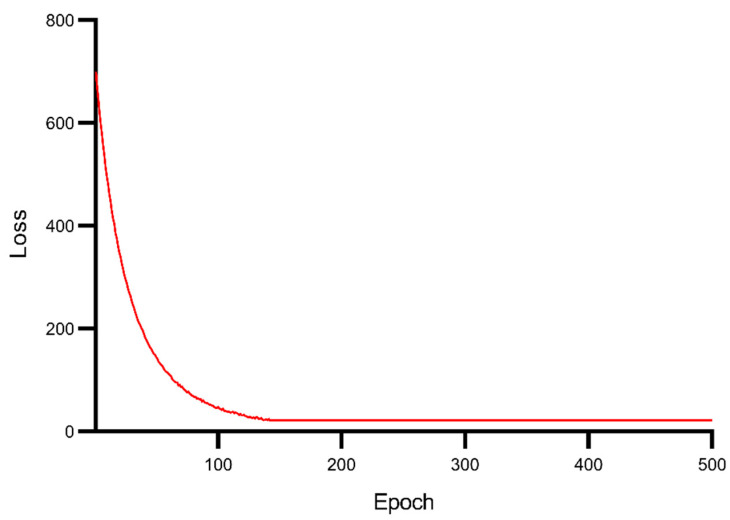
Convergence of BiLSTM model for predicting Baishuihe landslide displacement. In conclusion, the time series analysis method (EWMA) and the Double-BiLSTM dynamic landslide displacement prediction model can predict the displacement of the Baishuihe landslide well.

In addition to having good predictive performance, the BiLSTM model also has good convergence performance. It reaches convergence after 130 iterations, and the convergence process is shown in Figure 11.

## 4. Discussion

After comparing the results obtained with the mixed dynamic Double-BiLSTM model to the actual displacement values of the Baishuihe landslide, to verify the model prediction performance over other algorithms, this paper compared the Double-BiLSTM algorithm to other algorithms in terms of the trend or periodic displacement prediction. In the process of comparison, this paper considers the above four evaluation indices to evaluate objectively the gap between each model and the actual displacement data and then provides a further discussion and summary. In this paper, landslide displacement is decomposed into trend and periodic displacement components with the EWMA algorithm according to the time series analysis theory, so the prediction effects of trend and periodic displacement values are compared.

**Figure 12:**
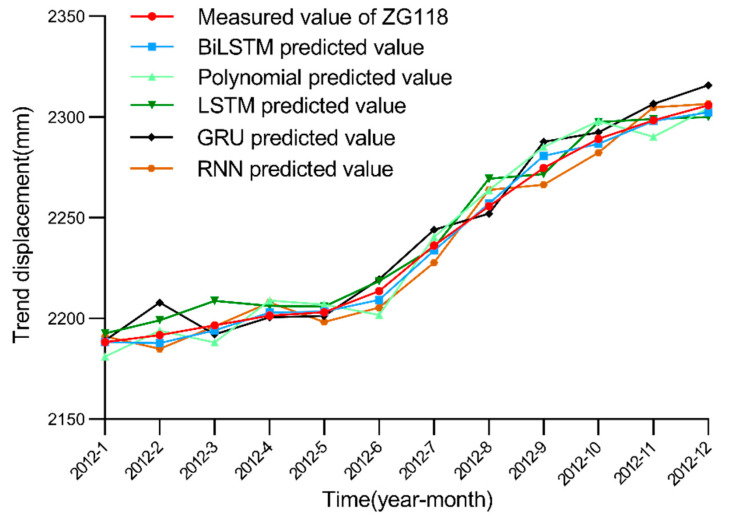
Measured and predicted trend displacement obtained with the BiLSTM, LSTM, GRU, RNN and polynomial models.

Because trend displacement is mainly affected by internal landslide factors, this paper directly applies the BiLSTM model to simulate and predict trend displacement. Compared to the traditional polynomial model 16,32], the BiLSTM model suitably processes time series data, can effectively record input data information and can steadily improve the model performance with increasing training data. After many experimental model training iterations, the number of nodes in the hidden layer of the BiLSTM model was set to 100, and the learning rate was set to 0.01. The parameters of the polynomial model in this paper are based on previous research [16,32], and the highest power is set to 3. A comparison of the trend displacement values predicted with the BiLSTM and other four models is shown in Figure 12.

**Figure 13:**
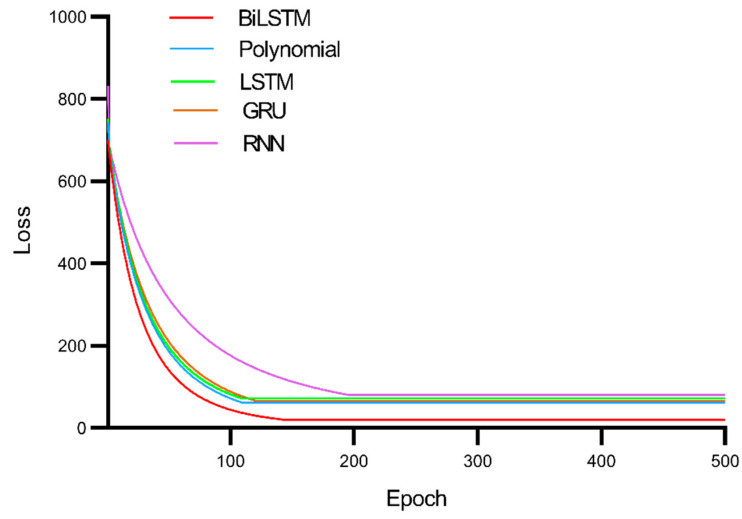
Convergence of BiLSTM, LSTM, GRU, RNN and polynomial models for predicting trend displacement.

Figure 12 reveals that the BiLSTM model is obviously superior to the other four models in landslide trend displacement prediction. Because landslide trend displacement also comprises typical time series data, the polynomial model simply treats trend displacement as ordinary data. RNN, LSTM and GRU also cannot process time series data as suitably as can the BiLSTM model. Figure 13 shows the convergence of these models. 

It can be seen from Figure 13 that the convergence speed of the polynomial model is fast, but the model error is large. The BiLSTM model has a slower convergence rate, but the error of the model is the smallest. This paper suggests that it is more important to sacrifice some convergence speed for a better prediction effect.

Table 2 records the performance evaluation index data of the BiLSTM and polynomial models in the trend displacement prediction of the Baishuihe landslide. Table 2 and Figure 12 indicate that the BiLSTM model yields smaller errors and that the overall model prediction performance is better than LSTM, RNN, GRU and polynomial models.

After trend displacement prediction, we predict and compare periodic displacement values of the Baishuihe landslide. Periodic displacement is influenced by many factors, and the occurrence is periodic and repetitive. Most of the landslides in the Three Gorges Reservoir area are affected by rainfall and reservoir water levels. To train the model better, we first employ the MIC, the information coefficient method, and select periodic displacement-related factors with high correlations. In this article, the MIC of the parameters is set to 0.6, and the associated factors of data generation introduced into the MIC model to calculate and select MIC results are greater than 0.3 [35]. Assuming that at time t, the landslide cumulative displacement is d(t); precipitation is p(t), and the reservoir water level is r(t); the MIC calculation results are listed in Table 3. Eleven factors are finally selected and incorporated into five models for training.

**Figure 14:**
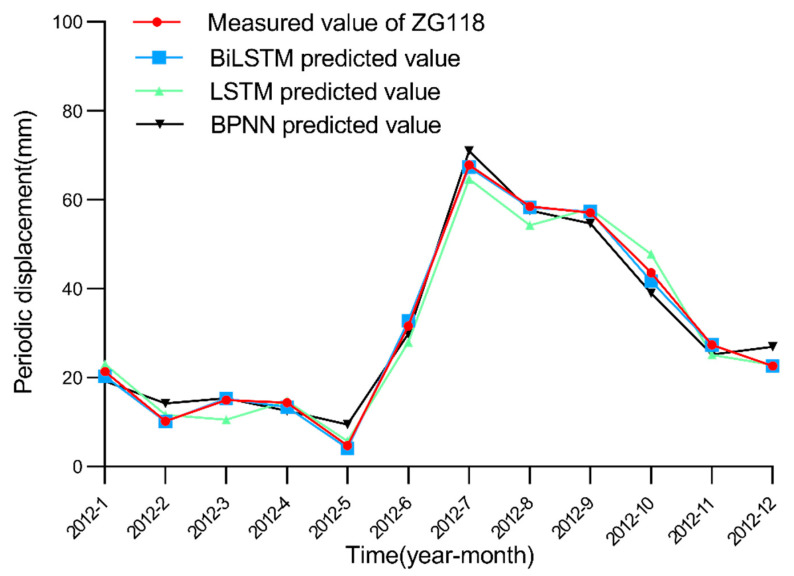
Measured and predicted periodic displacement values obtained with the BiLSTM, LSTM and BPNN models.

**Figure 15:**
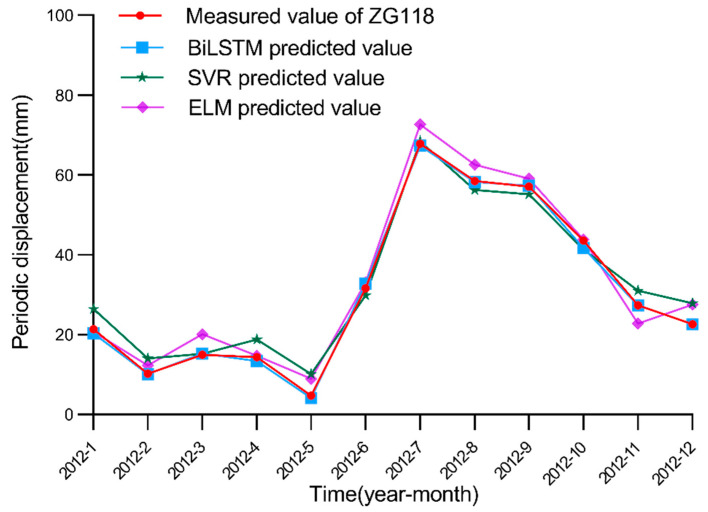
Measured and predicted periodic displacement values obtained with the BiLSTM, SVR and ELM models.

**Figure 16:**
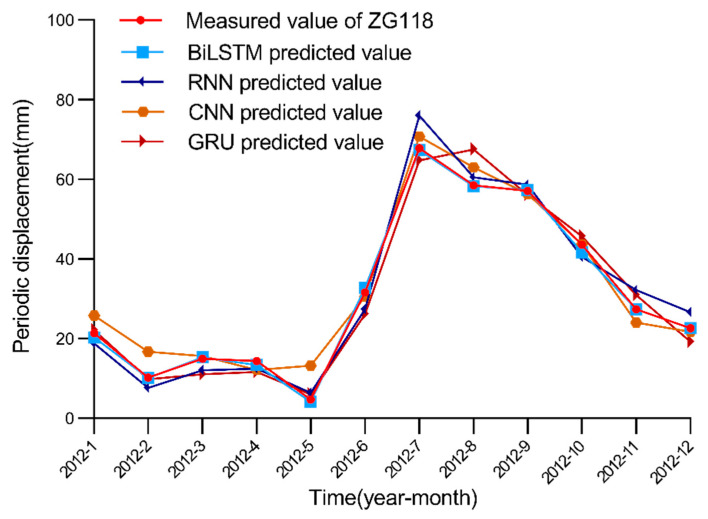
Measured and predicted periodic displacement values obtained with the BiLSTM, RNN, CNN and GRU models. Figure 14, Figure 15 and Figure 16 show that the BiLSTM model generates better prediction results in the periodic displacement prediction of the Baishuihe landslide. Figure 14, Figure 15 and Figure 16 reveal that the prediction performance of the LSTM and BiLSTM models is better than that of the other models because these two models suitably process time series data. The BiLSTM model considers the characteristics of the time series of the landslide displacement cycle. On the basis of considering the rainfall and landslide environments, the change in the reservoir water level during each cycle is cyclical and repeatable. Before training, the model not only considers current data but also considers future data to improve the performance of the displacement prediction model throughout the cycle. To illustrate the superiority of the BiLSTM model better, the evaluation index data of these machine learning algorithms in periodic displacement prediction are summarized in Table 4. The evaluation indices in the table indicate that the error between the predicted results obtained with the BiLSTM model and the actual periodic displacement data is minimal; the fluctuation in the model is relatively limited, and the model remains more stable.

After selecting 11 factors as the model input, we simulate the performance of each model in the periodic displacement prediction of the Baishuihe landslide considering these 11 factors. In this paper, after many experimental model training iterations, the parameter settings of the BiLSTM model are finally determined. We set the learning rate to 0.01 and the step size of the model to 12 according to the change period of rainfall and the reservoir water level, while the number of nodes in the hidden layer is set to 100. The BiLSTM model is compared to seven traditional machine learning models, namely, the LSTM model, ELM, SVR, RNN, CNN, GRU and back-propagation neural network (BPNN). For LSTM, GRU, RNN, ELM and BNPP models, we set the learning rate to 0.01; the number of nodes in the hidden layer is set to 100. For the CNN model, we set the channels of convolutional layer to be 12; the number of nodes in the fully connected layer is 100, and the activation function is Relu. For the SVR model, we set the kernel to be rbf, and the gamma is 0.01. The epoch of all models is 500. The simulated prediction results are shown in Figure 14, Figure 15 and Figure 16.

**Table 4:** Evaluation index of each periodic prediction model.

Models	MAE	MAPE (%)	RMSE	R2 (%)
BiLSTM	0.696	3.256	0.81	99.8
LSTM	2.285	9.893	2.285	98.1
BPNN	2.71	18.292	2.71	97.7
SVR	3.239	16.649	3.239	96.9
ELM	2.885	17.589	2.885	97.2
RNN	3.297	15.003	3.748	97.3
CNN	2.987	26.508	3.9	97.1
GRU	3.056	12.619	3.826	97.8

In order to display the model performance better and prevent the over-fitting phenomenon in the model training process, indicators were used to evaluate the training set and testing set of Baishuihe landslide data; the results are shown in Table 5 and Table 6.

The results of Table 5 and Table 6 show that the indices of the training set and testing set are similar when the BiLSTM model is used to predict the trend and periodic displacement. This indicates that the model has learned the general features of the data and has not taken the local features of the training set as the general features. The prediction performance of the model has no obvious change, and there is no fitting phenomenon.

Although the dynamic mixed landslide displacement prediction model proposed in this paper achieves a satisfactory performance in the Baishuihe landslide prediction, there remain improvements to be made. The use of the BiLSTM model to predict trend displacement and the BiLSTM model to predict periodic displacement produces more process parameters that should be adjusted during model training than are produced by the traditional polynomial, RNN, CNN, GRU and LSTM models, which increases the difficulty of model training. It is challenging to determine the parameters of the entire model to obtain the optimal state. The second deficiency is that the mixed proposed model has only been verified against the Baishuihe landslide but has not been applied in other regions or landslides, so the stability and accuracy of the model cannot be guaranteed. In the future, we will consider further model improvements to reduce the difficulty of the model parameter adjustment and improve the model prediction performance. In addition, the model will be applied in other areas that may produce landslides or other landslides to verify the feasibility of the proposed model better.

## 5. Conclusions

Landslide displacement prediction constitutes the key to landslide warning systems [14]. Better prediction of landslide displacement allows decision makers to make better decisions about whether the slope or the area is prone to landslides so as to warn people who live or work here and protect their health and safety. In previous studies, most landslide displacement prediction algorithms did not treat landslide displacement data as time series data, which resulted in certain limitations on the prediction performance of previous models. Considering that landslide cumulative, trend and periodic displacement data comprise typical time series data, a time series analysis method and Double-BiLSTM hybrid dynamic landslide displacement prediction model are proposed. In this paper, the EWMA-based method fully considers the time series characteristics of landslide data and decomposes landslide displacement into trend and periodic displacement components. Considering that trend displacement is mainly influenced by internal landslide factors, this paper employs the BiLSTM model, which suitably processes time series data to predict trend displacement. As the landslide displacement process is cyclical, previous and post landslide data can provide a certain reference function, and we apply the MIC method to select the factors influencing the high correlation to periodic displacement. The BiLSTM model parameters are trained on these data, and landslide periodic displacement is predicted. In this paper, actual landslide data pertaining to the Baishuihe landslide in the Three Gorges, China, over 108 months are selected in simulation experiments to verify the feasibility and accuracy of the proposed hybrid dynamic Double-BiLSTM landslide displacement prediction model. The trend and periodic displacement prediction results are compared to those obtained with previous algorithms or models. In the comparison process, four kinds of commonly considered prediction model performance indices are adopted, which verifies that the performance of the proposed model is notably better than that of the other landslide displacement prediction models. In conclusion, the time series analysis method and dynamic hybrid landslide prediction model proposed in this paper can achieve accurate landslide displacement prediction, effectively improve the judgment of common people and decision makers on landslide, reduce the suddenness of landslides and improve human welfare. This method can be applied in other landslide areas or other time series data prediction fields. 

## Figures and Tables

**Table 1:** Factors of model training for periodic displacement prediction.

α	Rainfall—MIC	α	Reservoir Water Level—MIC
0.05	0.324714915	0.05	0.751251529
0.1	0.324715703	0.1	0.751252363
0.15	0.324716778	0.15	0.751252991
0.2	0.324716832	0.2	0.751253684
0.25	0.324716885	0.25	0.751253792
0.3	0.324716872	0.3	0.751252125

**Table 2:** Evaluation index of each trend prediction model.

Models	MAE	MAPE (%)	RMSE	R2 (%)
BiLSTM	2.39	10.7	2.96	99.5
Polynomial	6.863	30.7	7.523	96.9
LSTM	4.94	20.2	5.942	97.9
GRU	5.843	26.2	6.924	97.2
RNN	6.282	28	7.126	96.9

**Table 3:** Factors of model training for periodic displacement prediction.

Factors	MIC	Factor	MIC
r(t-2)	0.551	r(t-1)	0.345
p(t-2)	0.438	p(t-1)	0.316
r(t-1) + r(t-2)	0.429	p(t-3)	p(t-3)
d(t-2)	0.417	p(t-1) + p(t-2) + p(t-3)	0.264
d(t-1) + d(t-2)	0.406	r(t-1) + r(t-2) + r(t-3)	0.248
d(t-1)	0.406	d(t-3)	0.228
p(t-1) + p(t-2)	0.362	d(t-1) + d(t-2) + d(t-3)	0.217
p(t)	0.350	r(t-3)	0.214
r(t)	0.349		

**Table 5:** Evaluation index of trend displacement.

Data	MAE	MAPE (%)	RMSE	R2 (%)
Training Set	2.31	11.2	2.87	99.1
Testing Set	2.39	10.7	2.96	99.5

**Table 6:** Evaluation index of periodic displacement.

Data	MAE	MAPE (%)	RMSE	R2 (%)
Training Set	0.712	3.198	0.86	99.3
Testing Set	0.696	3.256	0.81	99.8

## Data Availability

Restrictions apply to the availability of these data. Data were obtained from the National Cryosphere Desert Data Center/National Service Center for Speciality Environmental Observation Stations and are available from http://data.casnw.net/portal/ with the permission of the National Cryosphere Desert Data Center/National Service Center for Speciality Environmental Observation Stations.
